# Acute Disseminated Encephalomyelitis Post COVID-19 Pneumonia

**DOI:** 10.7759/cureus.34615

**Published:** 2023-02-03

**Authors:** Abdulaziz A Alqarni, Danya A Aljafari, Faris A Alzahrani, Abdulrahman A Alharthi, Madihah S Alhubayshi

**Affiliations:** 1 College of Medicine, King Saud Bin Abdulaziz University for Health Sciences, Jeddah, SAU; 2 Research and Development, King Abdullah International Medical Research Center, Jeddah, SAU; 3 Department of Medicine, Ministry of the National Guard-Health Affairs, Jeddah, SAU

**Keywords:** glasgow coma scale, edema, sars-cov-2, covid-19 pneumonia, acute disseminated encephalomyelitis

## Abstract

Acute disseminated encephalomyelitis (ADEM) is a monophasic condition characterized by inflammation of the central nervous system. Besides multiple sclerosis, optic neuropathy, acute transverse myelitis, and neuromyelitis optica spectrum disorder, ADEM is a primary inflammatory demyelinating disorder of the central nervous system. It is estimated that approximately three-quarters of cases of encephalomyelitis occur after an infection or immunization, where the onset of neurological disease is coincident with a febrile event. Here, we report an 80-year-old woman with coronavirus disease pneumonia who developed sudden onset of decreased level of consciousness, focal seizure, and right-side weakness. Magnetic resonance imaging (MRI) of the brain showed a multifocal hemorrhagic lesion with surrounding edema, suggesting ADEM. An electroencephalogram (EEG) revealed moderate generalized encephalopathy. The patient received alternating pulse steroids with plasma exchange for five days. Subsequently, her Glasgow coma scale score continued to decrease, and thus, she required inotropic support until she expired.

## Introduction

Acute disseminated encephalomyelitis (ADEM) is a rare disorder characterized by monophasic episodes of demyelination and inflammation after an infection or vaccination [1,2]. ADEM is prevalent in children and young adults; however, it has been reported in very few middle-aged and older patients [3-7]. In the acute phase, ADEM is characterized by multifocal neurological symptoms. MRI of the brain usually reveals demyelinating changes. However, various neurological complications have been described in patients with coronavirus disease (COVID-19), and it has been hypothesized that severe acute respiratory syndrome coronavirus 2 (SARS-CoV-2) might result in neurotropic behavior in some cases [8]. COVID-19 severity varies among patients, especially if they have comorbid diseases [9]. One important comorbidity that was found to be associated with poor COVID-19 prognosis was malignancy [9]. In chronic lymphocytic leukemia, the most common type of leukemia in adults, the severity of COVID-19 increases with age [10]. However, this has not been proven to affect mortality [10]. Despite the immune-mediated nature of ADEM, various clinical outcomes have been reported when patients are treated with intravenous steroids or immunoglobulins; however, no randomized clinical trials have been performed to establish treatment guidelines [11]. Herein, we report a patient with COVID-19 pneumonia who developed ADEM during hospitalization and ultimately died.

## Case presentation

**Figure 1:**
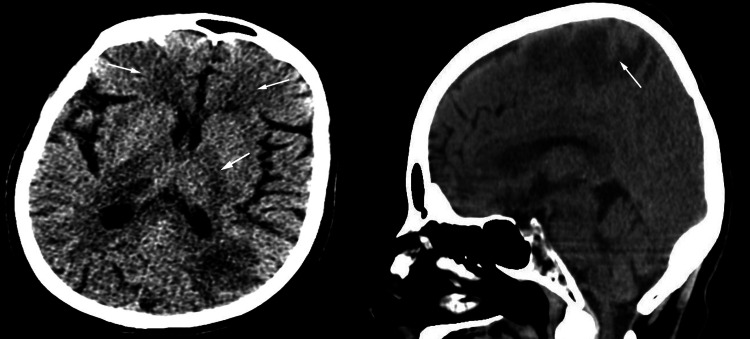
Brain computed tomography (CT) revealed multiple hypodense areas involving the bilateral frontal, right thalamic regions (a), and parietal lobe (b).

**Figure 2:**
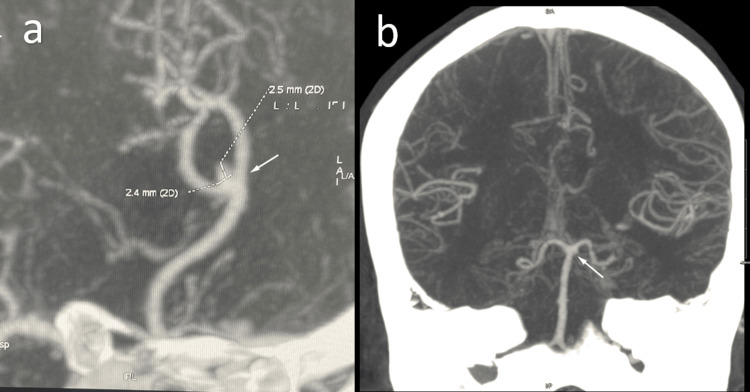
Brain computed tomography angiography (CTA) Small aneurysm at the bifurcation of the azygos anterior cerebral artery (ACA) measuring 2.5 mm in diameter (a,b)

An 80-year-old woman with a long-standing history of chronic lymphocytic leukemia experienced an influenza-like condition that persisted for two weeks. She tested positive for SARS-CoV-2 via a nasopharyngeal swab. Chest radiography (x-rays) revealed diffuse bilateral opacities, consistent with the diagnosis of COVID-19 pneumonia and which required treatment with antibiotics and dexamethasone. The patient initially required 2-3 L of oxygen and was started on tazocin and moxifloxacin. One week after admission, her white blood cell (WBC) count increased to 10.3x10^9/L, and she developed worsening hypoxia, which required a 60% high-flow nasal cannula. Meropenem and vancomycin were administered. Furthermore, her chest x-rays findings worsened, and her C-reactive protein (CRP) level started to increase. On day 15 of admission, the patient developed sudden onset decreased level of consciousness, focal seizure, and right-side weakness. Brain computed tomography (CT) revealed multiple hypodense areas involving the bilateral frontal, parietal, and right thalamic regions (Figure 1). She also received levetiracetam. Computed tomography angiography (CTA) revealed an accidental finding of a small aneurysm at the bifurcation of the azygos anterior cerebral artery (ACA) measuring 2.5 mm in diameter and minimal air-fluid level in the maxillary sinuses, representing acute sinusitis (Figure 2). Later, the patient was transferred to the intensive care unit and was intubated because of a decrease in consciousness level. Her Glasgow coma scale (GCS) score decreased from 13 to 6. Laboratory markers showed a worsening WBC count of 14 x10^9/L, and her blood cultures were negative. Her GCS score dropped further to 3, and she was started on a vasopressor.

**Figure 3:**
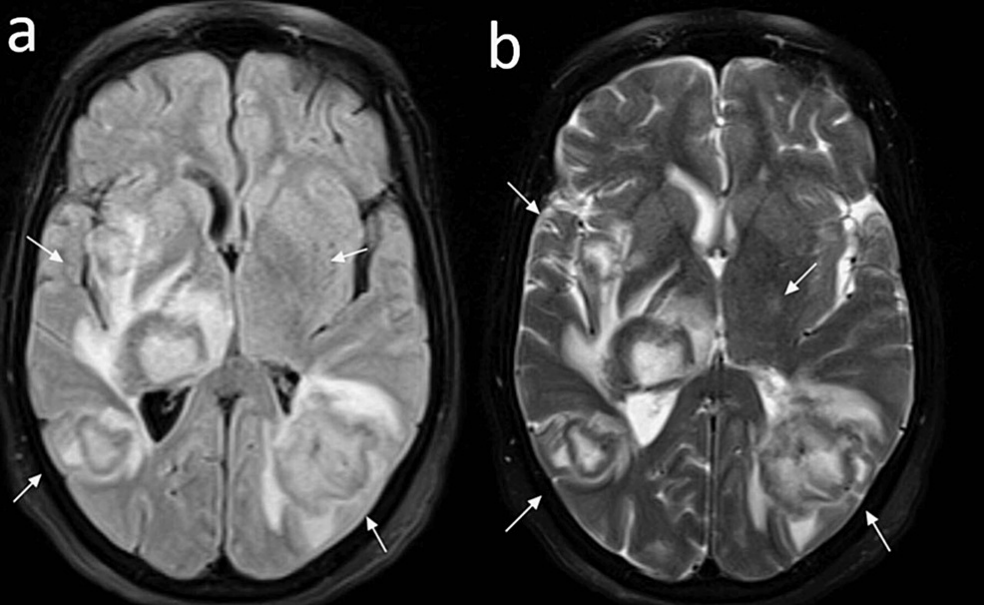
Brain magnetic resonance imaging Axial susceptibility-weighted imaging images showed multifocal non-confluent white matter lesions seen in both cerebral hemispheres having a central bright fluid-attenuated inversion recovery (FLAIR) signal intensity and peripheral dark FLAIR signal (a). Hemorrhagic components and restriction are seen as well on diffusion-weighted imaging (DWI) at the periphery of the lesions (b). Marked perilesional edema is noted. Involvement of the right thalamus with surrounding edema and some compression of the adjacent midbrain and the third ventricle causing mild entrapment of the frontal horn of the right lateral ventricle. This pattern of multifocal lesions with surrounding edema has been described in COVID post viral infection brain hemorrhagic lesions. Appearance can be like hemorrhagic acute disseminated encephalomyelitis.

Brain MRI showed a multifocal hemorrhagic lesion with surrounding edema, suggesting ADEM (Figure 3). The patient received alternating pulse steroids alternating with plasma exchange for five days. Twenty-two days post-admission, the patient’s GCS score remained low at 5/15 off sedation. She also had two spikes of fever (38.2°C was the highest), and her WBC count increased to 31.3; however, her CRP level improved. The day after, the patient’s GCS dropped to 3/15, and she was put on pressure support ventilation; however, was not triggering the ventilator and she had no cough or gag reflex. Her pupils measured 3 mm and were sluggish bilaterally, and she was being treated with dehydrating measures, mannitol, and hypertonic saline 3%.

**Figure 4:**
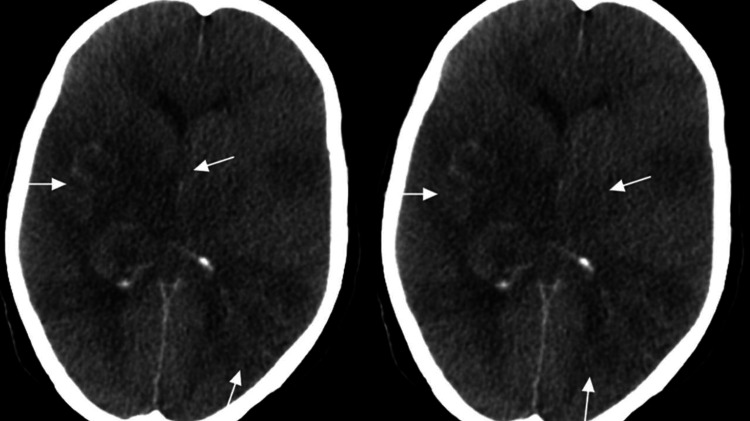
Brain computed tomography (CT) CT brain revealed significant interval worsening of the vasogenic edema with interval development of cytotoxic edema causing a mass effect with cerebellar tonsillar herniation, bilateral uncal herniation, and possible venous sinus thrombosis.

A static CT brain revealed significant interval worsening of the vasogenic edema with interval development of cytotoxic edema causing a mass effect with cerebellar tonsillar herniation, bilateral uncal herniation, and possible venous sinus thrombosis (Figure 4). High increased intracranial pressure (ICP) measures were continued, after which she developed hypernatremia and was started on free water 200 mL. Twenty-six days post-admission, the patient required high inotropic support, and a few hours later, she developed asystole and was declared dead after the failure of cardiopulmonary resuscitation.

## Discussion

SARS-CoV-2 infection was declared a global pandemic by the World Health Organization on March 11, 2020 [12]. COVID-19 is primarily characterized by symptoms of respiratory distress and has been associated with several neurological manifestations during hospitalization. There is no clear evidence about how SARS-CoV-2 affects the brain and spinal cord. Angiotensin-converting enzyme-2 receptor (ACE2)-mediated neuronal and glial cell injuries are widely considered to be the mechanism causing neurological injury, resulting in demyelinating lesions in the brain and spinal cord [11]. ADEM is the most severe manifestation of demyelinating inflammatory conditions caused by COVID-19. Encephalopathy of non-specific onset can be associated with changes in behavior or changes in the level of consciousness, leading to low GCS, with or without fever. The most common neurological deficits are polyfocal and include visual field deficits and hemiparesis. In addition, a history of viral infection supports this diagnosis [13].

Radiologically, MRI is considered the modality of choice of ADEM which is characterized by widespread and asymmetrical lesions throughout the white and gray matter of the brain. Lesions typically appear hyperintense on T2-weighted and fluid-attenuated inversion recovery (FLAIR) imaging [14]. ADEM can also be observed on MRI as a hemorrhagic lesion [6]. Cerebrospinal fluid (CSF) analysis should also be performed to assess for the presence of elevated protein levels, which were noted in all three patients mentioned in a case series of ADEM resulting from COVID-19 [15]. Regarding the use of CT, previous studies have reported unremarkable findings in patients. However, our patient had significant vasogenic edema with interval development of cytotoxic edema, causing a mass effect with cerebellar tonsillar herniation and bilateral uncal herniation.

To prevent prolonged hospital stay and permanent neurological deficits, early treatments should be initiated [13]. Intravenous corticosteroids remain the first-line treatment of choice for these patients [13]. According to a case series that included three patients with the same condition, one patient was administered only intravenous corticosteroid and showed great improvement, while the other two patients showed only mild improvement; therefore, intravenous immune globulin (IVIG) was added to the treatment [15]. Our patient received pulse steroids alternating with plasma exchange for five days; however, because of her severe condition and age, her condition continued to worsen, and she subsequently died.

## Conclusions

Based on our case findings, ADEM should be suspected in patients with COVID-19 who present with sudden neurological changes such as weakness, behavioral changes, or altered levels of consciousness. MRI and CSF analysis should be performed for these patients to aid in confirming the diagnosis, and appropriate treatment should be initiated early.
